# Exposure to humans and task difficulty levels affect wild raccoons (*Procyon lotor*) learning

**DOI:** 10.1093/beheco/arae046

**Published:** 2024-06-05

**Authors:** Louis Lazure, Robert B Weladji

**Affiliations:** Biology Department, Concordia University, Montréal, Québec, Canada; Conservation and Research Department, Zoo de Granby, Granby, Québec, Canada; Biology Department, Concordia University, Montréal, Québec, Canada

**Keywords:** behavior, cognition, foraging, innovation, memory, problem-solving

## Abstract

Cognition helps wildlife exploit novel resources and environments. Raccoons (*Procyon lotor*) have successfully adapted to human presence, in part due to their cognitive abilities. However, interactions between humans and wildlife can create conflict. A better understanding of the raccoon’s behavioral flexibility and learning ability could mitigate some conflicts. Our objective was to evaluate wild raccoons learning in contexts varying in terms of exposure to humans (recreational and preservation zoning within protected areas) and task difficulty. Learning can be evaluated over multiple exposures to a cognitive task. Across three years of experiment, we employed 2 food extraction tasks to gauge the change in problem-solving performance over trials. This assessment considered the success probability (the number of successful trials divided by the total number of trials) and the time taken to solve the puzzles. We also looked at the effects of 2 behavioral traits, exploratory diversity and persistence. We found strong evidence for learning over consecutive trials in terms of improved success probability. Improvement in terms of success probability and solving time was more pronounced with the initially easier task. We detected an increase in success probability over trials only in the recreation zones, and there was no evidence of an effect of behavioral traits. The improved performance attributed to learning was also maintained over consecutive years. We provide additional evidence that raccoons can learn how to solve a problem, resulting in a more effective solution in consecutive trials. Finally, we consider the management implications of dealing with raccoons accessing anthropogenic resources.

## Teaser

Raccoons rely on their learning ability to take advantage of novel resources in their environment. Using two problem-solving tasks, we tested wild raccoons living in different zones of protected areas. Raccoons indeed increased their success probability over consecutive trials. Those who lived in the zone with higher human exposure also improved solving time over trials. Raccoons retained their success probability over the winter season, showing signs of long-term retention, or memory, of learned solutions.

## Introduction

Species in the order Carnivora are often involved in human‒wildlife conflicts (Sillero-Zubiri and Laurenson 2001; Treves and Karanth 2003; Carter and Linnell 2016; Bergstrom 2017; Expósito-Granados et al. 2019; Lozano et al. 2019). Raccoons (*Procyon lotor*) are medium-sized mesopredators (Buskirk and Zielinski 2003; Prange and Gehrt 2004; Prugh et al. 2009; Glas 2016). They are generalists in terms of diet and habitat (Lotze and Anderson 1979; Bozek et al. 2007) and are well adapted to anthropogenic landscapes such as rural and urban areas (Prange et al. 2004; Bozek et al. 2007; Hadidian et al. 2010; Daniels et al. 2019). Their high manual dexterity relative to other Carnivores (Iwaniuk and Whishaw 1999) might also explain their success in urban areas. Raccoons are often vilified as a pest species, despite the severity of conflict being relatively benign compared to other cases of human‒wildlife conflicts (Pettit 2010; Pacini-Ketchabaw and Nxumalo 2016; Barrett et al. 2019; Justice 2021). Reasons to want to reduce the contact rate between humans and raccoons include reduced disease transmission risk, unwanted habituation, damage, and interaction with pets (Rosatte 1998; Beasley and Rhodes 2008; Hadidian et al. 2010; Prescott 2011; Bateman and Fleming 2012; Glas 2016).

The behavior of raccoons in conflict with humans is poorly understood, and data on their responses to different control strategies is insufficient (Curtis and Hadidian 2010). The need for conservation actions and their efficacy are tightly linked to wildlife behavior and cognition (Greggor et al. 2014; Goumas et al. 2020). Understanding and considering learning is essential in devising successful repellents and conditioned-taste aversion mitigation methods (Greggor et al. 2020). A manager could theoretically take advantage of a nuisance animal’s learning ability by conditioning it to adopt an appropriate behavior (Greggor et al. 2014). The relatively high learning abilities exhibited by raccoons make them good candidates for conditioning (Snijders et al. 2021).

Learning is a difficult concept to define but is characterized by a change in cognitive state resulting from experience (Pearce 2008; Shettleworth 2010) that persists for a certain time (Domjan, 2000). There are multiple variants of learning, and this study is interested in operant learning, where the animal learns a predictive relationship between an action and an outcome (Shettleworth, 2010; Griffin et al. 2015). Sequentially, it is the ability to acquire, consolidate, and retrieve information (Papini and Torres 2017). Learning permits the integration of innovations into the behavioral repertoire (Ramsey et al. 2007). Memory of learned knowledge may fade over time (Shettleworth 2010), for example, during prolonged periods of disuse such as over winter (Mateo and Johnston 2000; Mateo 2010). Learning is a form of phenotypic plasticity and can be subject to selective forces (Dukas 2013). Unambiguous cues, such as the presence of food in proximity to humans and rapid feedback, make learning especially susceptible to selective pressure (Sih 2013). As a cognitive process, learning ability differs among individuals in a consistent manner, akin to behavioral personality traits (Sih and Del Giudice 2012). Learning is highly contextual, and environmental characteristics can shape cognitive performance, which enables wildlife to adapt to new challenges (Greggor et al. 2014; Owen et al. 2017; Chow et al. 2021b), especially in a rapidly changing human context (Sih 2013; but see Greggor et al. 2019). Through learning mechanisms within its lifespan, an individual will adjust its behavior to avoid risks and take advantage of opportunities (Snell-Rood 2013; Sol et al. 2013; Fehlmann et al. 2021).

Learning is almost exclusively studied through experiments because naturalistic observations are blind to the experience of the individual (Domjan 2000). Learning has been studied more extensively in laboratory settings, but there is still work to be done in ecologically relevant contexts (Thornton et al. 2014; Greggor et al. 2020). Physical challenges, such as the puzzle box paradigm, allow researchers to assess one’s performance in cognitive tasks (Reader et al. 2016; Washburn et al. 2017; Barrett et al. 2019; Daniels et al. 2019; Johnson-Ulrich et al. 2020; Benson-Amram et al. 2022). Puzzle boxes are ecologically relevant because raccoons have learned to take advantage of anthropogenic food resources, often overcoming similar types of physical challenges to obtain rewards (Bateman and Fleming 2012; MacDonald and Ritvo 2016; Barrett et al. 2019; Daniels et al. 2019). Food is a central problem in human‒wildlife conflicts (Donaldson et al. 2012), and the most biologically relevant and sensory salient experiences are learned faster (Shettleworth 2010). Problem-solving tasks are often used to study learning, and an animal with high cognitive abilities could perform well in both (Griffin and Guez 2014); however, there is no clear relationship between problem-solving and learning (Seed and Mayer 2017). Using more than one test allows us to assess convergent validity: a “good” performance at one test should be reflected in the other as well (Boogert et al. 2018). Reproducing experiments with different populations in various natural settings are also important to study the effect of the environment on cognitive abilities (Healy and Rowe 2014; Morand-Ferron et al. 2016; Pritchard et al. 2016; Horn et al. 2022; Johnson-Ulrich et al. 2022; Thornton and Truskanov 2022).

Learning is part of a raccoon’s foraging strategy (Dalgish and Anderson 1979) and is also involved in food extraction tasks (Daniels et al. 2019). We hypothesize that successful raccoons will exhibit operant learning and predict a relationship between trial numbers and success probability, time to solve, and exploratory motor diversity. In addition to trial number, we will test for the effect of the puzzle type and the human exposure (recreation versus preservation zones). We predict that learning improves faster (higher increase in success probability and reduction in solving time over consecutive trials) with the easier task and that it will be more important (higher success probability increase and quicker solution over consecutive trials) in recreation zones compared to preservation zones. Finally, we hypothesize that the time period between field seasons has a negative effect on the retention of learning, or memory. We predict that the first trial the following year will be less successful than the last ones from the previous year.

## Methods

### Field work

We conducted experiments in 3 protected areas located in southern Québec, Canada (all located around latitude 45.6° N and between longitude 72.6° and 75.2° W): Plaisance (28 km^2^), Îles-de-Boucherville (8 km^2^) and Yamaska (13 km^2^) national parks. These parks are considered to have a high density of raccoons, causing “severe” nuisance problems (Dellarosa 2012; Denis 2017). All these parks are relatively small, encompassed in mostly urban or agricultural territories, and border large bodies of water (river or lake). Two site categories are studied based on management zoning: intensive recreation and preservation zones. Recreation zones were defined by the presence of camping sites, vehicular circulation at low speed (< 20 km/h), campfires, dumpsters, and a mosaic of ground cover (gravel or paved roads, parking lots, forests, open fields, buildings, and playgrounds). Preservation zones were strictly accessible to the public by walking and biking trails, with extensive forest cover. We ran the experiments for three summers (earliest-latest dates: May 31‒Sept. 14) between 2019 and 2021. Plaisance Park was not visited in 2021 because of its lower raccoon activity compared to the 2 other parks and its lower accessibility.

We used species-specific baits, but all wildlife could interact with the devices (we only recorded eight interactions by stripped skunks in addition to raccoons). The experiments were non-invasive; animals voluntarily approached the apparatus and left. This ensured that only motivated animals participated. Raccoons were trap-shy, and although tested, capture-marking did not prove efficient in identifying individuals. Raccoons were identified solely by LL through careful observations of the video footage, based on their size relative to the puzzles, body characteristics (fur, tail, limbs), marking when available, and scars and injuries, in a similar manner to Chow et al. (2021a) with Eastern gray squirrels (*Sciurus carolinensis*). Juveniles were excluded because they showed very little initiative and were impossible to tell apart from the videos, often interacting together with the devices, therefore creating confusion in tracking one individual at a time. It was also impossible to identify an individual as a juvenile for 1 yr and as a grown adult for consecutive ones. To increase our confidence in the identification process, we conducted an intra-rater reliability test (Cohen’s kappa) on a small subset of recordings from the site with the highest activity level (Îles-de-Boucherville, recreation zone). We obtained an 87% agreement (*κ* = 0.851) corresponding to an “almost perfect” agreement (Landis and Koch 1977).

**Fig. 1.**
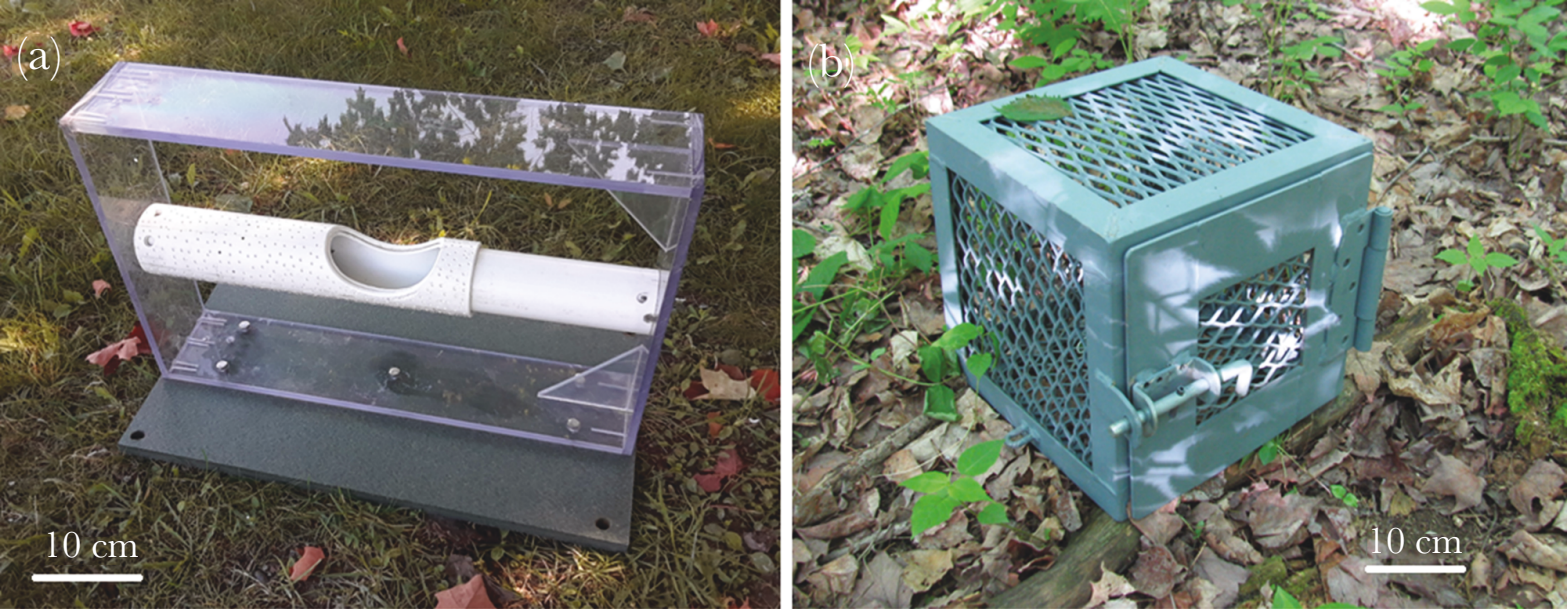
Cognitive puzzle boxes used to test problem-solving and learning in wild raccoons. (a) Tube and (b) Box puzzles.

We used 2 puzzle box tasks to test problem-solving abilities (Fig. 1). Raccoons trying to open food containers (e.g. plastic boxes, bags, and bottles) is a common occurrence when they are exposed to humans, making this task contextually relevant (Knight 2022). The first puzzle we used (hereafter, the Box) is similar to the model used with carnivore species in other studies (Benson-Amram and Holekamp 2012; Benson-Amram et al. 2016). Using the same type of puzzle facilitates the comparison of our results to similar experiments (Krasheninnikova et al. 2020). The Box measured 30 cm on each side and was made from steel mesh. To solve this problem, a raccoon had to slide a latch and pull on a door. The second puzzle (hereafter, the Tube) consisted of two horizontal plastic tubes (50 cm long in total and 7 cm in diameter), one sliding over the other. It required the animal to slide and turn the outermost tube to align two holes (approximately 5 × 10 cm) and access the food in the inner tube. Both puzzles necessitated 2 consecutive actions that can be performed with the paws, mouth, or muzzle of the animal.

### Video analysis

We considered the puzzle to be solved when a raccoon opened it enough to have direct access to the food with its paw, even if it did not immediately reach in and consume the reward. An attempt began when an animal approached within one body length of the box and ended when the animal moved more than a body length away from the puzzle or when it opened the puzzle. A trial included all attempts at opening a puzzle within a single night, so the trial ended when the last attempt ended. We recorded interactions with night vision cameras (Argus 2, Reolink, Hong Kong) set up 3‒4 m away (see Supplementary Material for an example with each puzzle). We quantified cognitive performance in problem-solving ability from the videos. We used 2 response variables to quantify problem-solving efficacy: (1) success (binomial) when the subject opens the puzzle (or not) to have direct access to the bait and (2) time to solve (continuous), which is the cumulative time from the first attempt until the puzzle opening within a trial. Two discrete terms representing behavioral traits were included in our models: persistence is the sum of all attempts within a trial, including the one when the puzzle is solved, and exploratory diversity is the number of unique behaviors directed at the puzzles. We calculated exploratory diversity in a similar manner to previous studies (Benson-Amram and Holekamp 2012; Benson-Amram et al. 2013, 2014; Johnson-Ulrich et al. 2018; Daniels et al. 2019). The exploratory diversity score accounts for behaviors without contact that allow the gathering of information (circling around the puzzle and sniffing), as well as behaviors with physical contact (Table 1).

**Table 1.** Ethogram of observed behaviors expressed by raccoons interacting with the puzzle boxes, used in quantifying exploratory diversity, and frequency observed among all interactions (*n *= 948).

Behavior	Description	Frequency (%)
Bite	Open mouth and close teeth around a puzzle box feature	1
Circle	Move around puzzle box within arm’s length	9
Climb	Raise body vertically along the puzzle box	8
Dig	Use paws to dig around the puzzle box	3
Lick	Open mouth and move tongue onto a puzzle box feature	1
Pull box	Use limbs to move puzzle box toward self	5
Pull knob	Use mouth or paws to move knob of door solution toward self	3
Push with arms	Use limbs to move puzzle box away from self	1
Push with head	Place head against puzzle box and move forward	1
Raise	Use nose or paws to move ledge of window solution up	<1
Reach with paw	Place paw through puzzle box to retrieve food reward	6
Slide	Use mouth or paw to move knob of slide solution to the left	6
Sniff	Draw in air through the nostrils to detect a scent	33
Stand on	Position body on top of the puzzle box	4
Touch	Place paw on a puzzle box feature	19

*Source*: Adapted from Daniels et al. (2019).

### Data analysis

We performed a generalized linear mixed-effect model (GLMM) to examine how success changed over successive trials. We used a binomial distribution with the logit function and included the fixed covariates zone and puzzle type in interaction with trials. Individual and year were included as random effects. To assess the effect of trial number on solving time, we ran a GLMM using only successful trial data. We used a Gamma distribution with the log function. We explored interactions between trials and zones, as well as between trials and puzzle types. Year and ID were included as random terms, and the model was optimized using the Nelder-Mead optimizer from the R package “nloptr” (Nelder and Mead 1965; Johnson 2014).

To see if persistence (number of attempts) at a task changed over trials, we performed a GLMM with a Poisson distribution and log link function, controlling for repeated measures within an individual by including ID as a random term. We also calculated the success probability and mean number of attempts on unsuccessful trials (as a proxy of persistence) for each individual and tested for a correlation with a Kendall rank correlation coefficient. To see if exploratory diversity changed over trials, we performed a GLMM with a Poisson distribution and log link function, controlling for repeated measures within an individual by including ID as a random term.

To assess the effect of year, we used a log-likelihood ratio test, comparing a model with year as a random effect to one without, while keeping the fixed effect structure constant (lmtest package; Zeileis and Hothtorn 2002). We also performed McNemar’s Test (with continuity correction) to determine if the proportions of success significantly differed when matching pairs of subjects (Fagerland et al. 2014) at their last trial of the year compared to the first of the next year. We used the program R to run all statistical analyses (4.2.3, R Core Team 2023).

## Results

We recorded 331 trials from 67 individual raccoons. The minimum number of trials (by individual, by puzzle) was 2, the maximum was 10, and the mean (± SE) was 2.7 ± 0.1. Thirty-five individuals interacted with the same puzzle 3 times or more. Not all individuals interacted with both puzzles. The percentage of raccoons that did not solve the puzzles was 66% for the Box, 27% for the Tube, and 17% for neither. We recorded 238 trials by 48 individuals in recreation zones and 172 trials by 49 individuals in preservation zones.

**Fig. 2.**
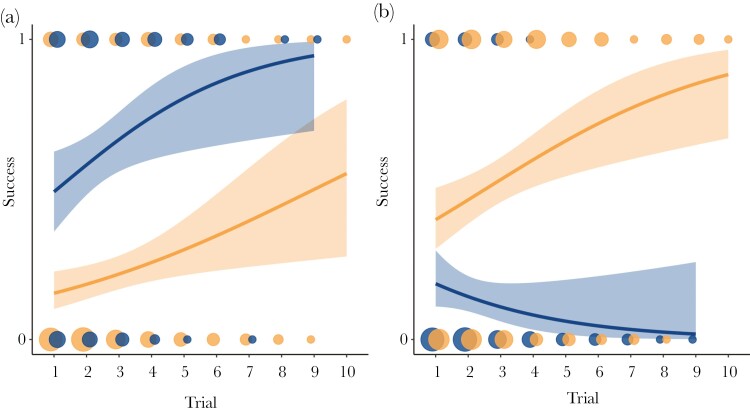
Logistical regression lines (mean ± 95% CI) of the success probability of wild raccoons trying to solve cognitive puzzles on consecutive trials. a) Comparison between the Tube puzzle (dark blue) and the Box puzzle (light orange). b) Comparison between raccoons in the recreation zone (light orange) and the preservation zone (dark blue). Points are dodged horizontally to avoid overlap.

The data revealed a positive relationship between success probability and trial number (*β* = −0.798, CI = −1.180, −0.416, *P < *0.001). Overall, the probability of success increased by 4% in each consecutive trial. There was very strong evidence that the relationship between success probability and trial number differed with puzzle type (*β* = 0.752, CI = 0.475, 1.028, *P < *0.001), with an overall success probability almost twice as high (1.95 times) on the Tube (Fig. 2A). There was also very strong evidence for a relationship with zone (*β* = 0.921, CI = 0.598, 1.244, *P < *0.001), with an improvement in consecutive trials from raccoons in the recreation zone but a slight negative trend in the preservation zone (Fig. 2B).

**Fig. 3.**
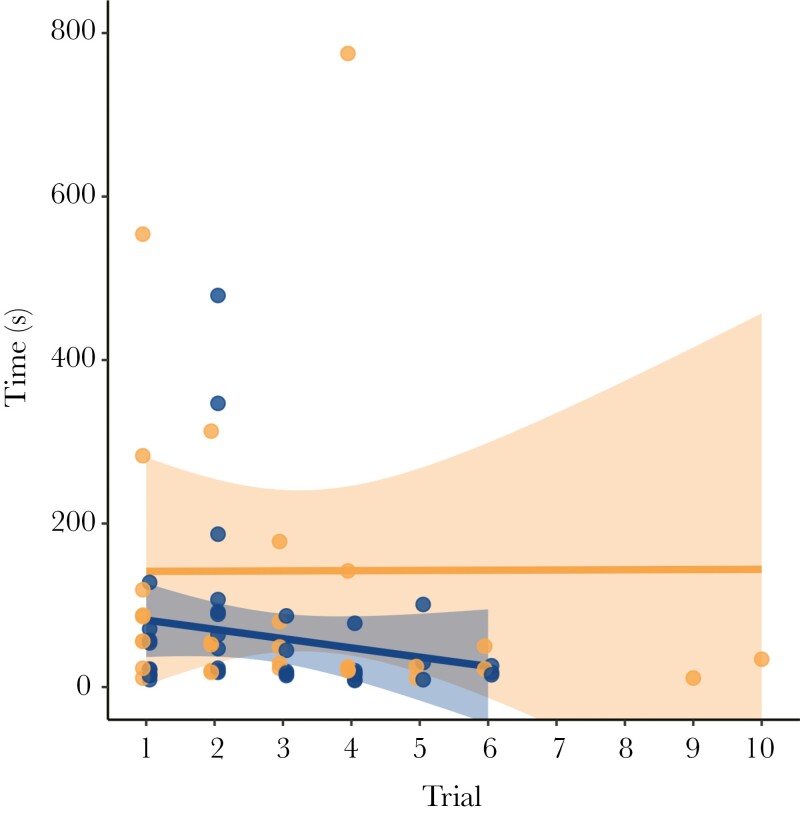
Linear regression line (mean ± 95% CI) of time (s) to successfully solve cognitive puzzles by wild raccoons over consecutive trials. Comparison between the Box puzzle (light orange) and the Tube puzzle (dark blue). One data point for the Box puzzle is not shown but was included in the analysis (coordinates 5, 1297). Points are dodged horizontally to avoid overlap.

The solving time ranged from 2 s to 36 m 46 s. We only included 72 successful trials in the time model. There was no evidence that the number of trials was related to time to solve (*β* = 0.131, CI = −0.346, 0.609, *P *= 0.589). There was, however, very strong evidence that there is an interaction between trials and both puzzle types (*β* = −0.264, CI = −0.389, −0.139, *P < *0.001), but little evidence of such interaction with zone (*β* = −0.189, CI = −0.636, 0.258, *P *= 0.406). The linear regression slopes indicate a decrease in solving time of −11.3 s per trial with the Tube and an increase of 0.3 s per trial with the Box (Fig. 3). Although there was little evidence to be significant, we see a slight decrease in time to solve in the preservation zone but almost none in the recreation zone.

Sniffing represented the most common behavior scored using the ethogram (33%, Table 1). There was no evidence that exploratory diversity was associated with trials (*P *= 0.665). This remained true when we only looked at successful trials (*P *= 0.928). The number of attempts also did not vary on successive successful trials (*P *= 0.222). There was weak evidence that there is a negative correlation between an individual mean number of attempts and mean success probability (tau = -0.192, *P* = 0.051).

**Fig. 4.**
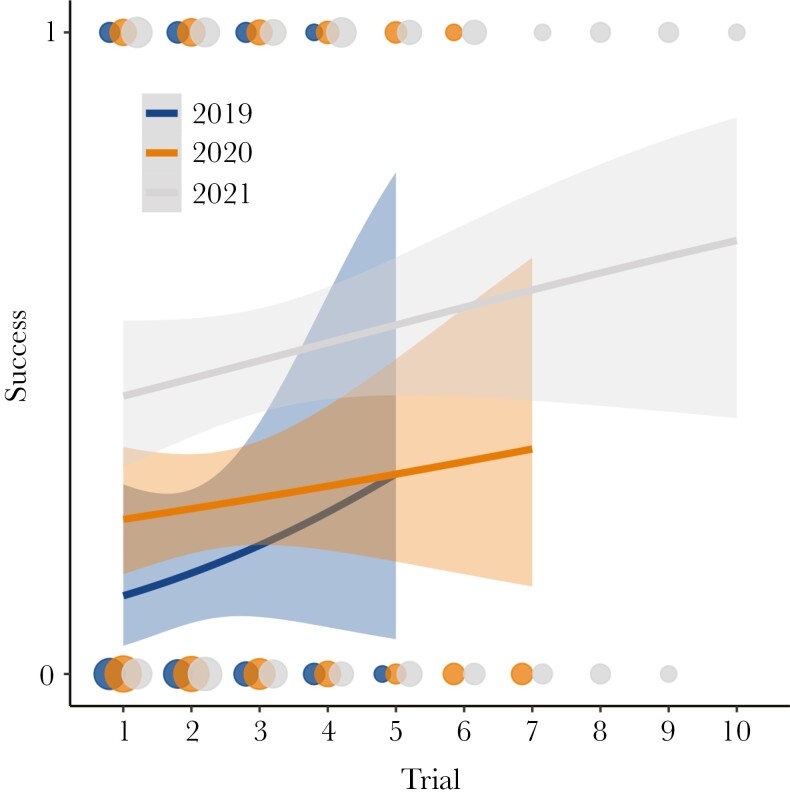
Logistic regression line (mean ± 95% CI) of success of wild raccoons taking part in cognitive tasks over consecutive trials in three separate field seasons (2019–2021, see legend). Points are dodged horizontally to avoid overlap.

Few individuals participated in consecutive years (*n* = 16). The mean success probability increased over trials and increased year after year (Fig. 4). Models differed significantly with or without year as a random factor (log-likelihood ratio test: χ^2^ = 15.5, *P* = < 0.001). Comparing the last trial from a given year to the first trial of the next year, we found no evidence of a change in the proportion of success (χ^2^ = 0.8, *P *= 0.371). The mean number of days between these trials in different seasons was 348 ± 7, with one individual interacting with the same puzzle at a two-season interval.

## Discussion

We tested the learning ability of wild raccoons by exposing them to 2 cognitive tasks on multiple nights. Similar to other studies on animal learning, food proved to be an effective positive reinforcement for raccoons (Galois 1996; Daniels et al. 2019; Morton 2021; Stanton et al. 2021, 2022). Raccoons demonstrated learning capabilities over consecutive trials by improving their success probability. This is indicative of the raccoons’ learning capacity, as previously demonstrated with different experiments (Dalgish and Anderson 1979; Galois 1996; Daniels et al. 2019; Stanton et al. 2022). This result could support the idea that generalist species have high innovative potential and learn fast (Henke-von der Malsburg et al. 2020). The raccoon’s ability to learn indicates a natural predisposition that has evolved over time (Shettleworth 2010; Fawcett et al. 2012), reflected in the cellular architecture of their brains (Jacob et al. 2021).

In the initial trial, the two puzzles were not solved at the same rate or speed (Fig. 2A); the Box was more challenging to learn to solve than the Tube. The improvement in performance (over consecutive trials) was different in terms of success probability and solving time between the two puzzles. This shows that task difficulty can constrain learning. Methodologically, it highlights the importance of using different tasks to quantify learning ability.

There was also an effect of the zone on the problem-solving success probability. Raccoons in the recreation zones, which were exposed to anthropogenic food resources, tended to improve their success probability over trials, while the performance of raccoons in preservation zones decreased (Fig. 2B). Anthropogenic landscapes and human routine, as observed in the recreation zone, provide more predictable resource availability (Griffin et al. 2017), and some predictability in the environment increases the benefit of learning and might select for enhanced learning (Dunlap and Stephens 2009; Mettke-Hofmann 2014; Griffin et al. 2017). Furthermore, Anderson (2020) found that relative brain size was higher in urban raccoons compared to rural ones, showing that further environmental differentiation can affect cognitive phenotype (Fawcett et al. 2012).

There was also no indication that raccoons lost acquired knowledge between seasons. Interestingly, they seemed to perform as well, in terms of success probability, toward the end of a given season and the start of the next, basically continuing from the point where they left (Fig. 4). This is indicative of long-term retention of learned solutions or memory (Papaj et al. 2019; Kirkpatrick and Hall 2022). Our study demonstrated that raccoons have the ability to retain foraging-related information for extended period of time, with their retention time spanning many months and averaging close to a full year. In previous experiments, raccoons retained their problem-solving knowledge over many months as well: up to 147 days (Cole 1907) and up to 286 days (Davis 1907). Davis (1907) extrapolated that problem-solving ability was preserved for more than a year. The memory window varies considerably between species, individuals, and within an individual’s own age and state (Dunlap et al. 2009).

Exploratory diversity remained the same over trials, thus we did not support the inhibitory control hypothesis. Daniels et al. (2019) found that raccoons improved their performance both in terms of speed of solving and by being more selective. A reduction in exploratory diversity by removing unnecessary behaviors would be indicative of inhibitory control (Reader et al. 2016). Inhibition is a cognitive mechanism of rejecting a behavior while favoring an alternative (Hauser 1999). This implies that there is a behavioral cost (time or energy) to sampling many behaviors, which might not be significant in our study context (Papaj et al. 2019). The number of attempts did not change between trials. Raccoons, being described as ‘stubborn’, present high levels of persistence, which positively correlated with success in an experiment by Daniels et al. (2019). We did not find such a correlation, with more persistent individuals not being more successful. In the context of this study, maybe there was less cost in giving up due to the relative abundance of other food sources in the environment.

The learning ability of raccoons, especially in human-altered habitats, presents a challenge when it comes to managing conflicts, but it can also be an opportunity (Schulte 2016; Barrett et al. 2019). In our experiment, we utilized a barrier in the form of puzzles to restrict access to food for the raccoons. Our findings revealed that as the difficulty of the task increased, the success probability decreased and the learning process was slower for the raccoons. Practically, for someone who wants to block access to a resource by raccoons, it is probably worth investing in a barrier or locking device that involves more complex actions from the start instead of trying cheaper, simpler options. Learning can be based on different sensory inputs; odors, in particular, can affect learning processes (Schulte 2016). Conditioned taste aversion (CTA) is an approach that creates an association (learning) between a sensory cue and an aversive stimulus (Shivik et al. 2003; Snijders et al. 2021; Kirkpatrick and Hall 2022). Because we showed learning ability and relatively long-term retention in the raccoons, it opens the door to the mitigation of conflict through deterrent stimuli (Schakner and Blumstein 2013). Some authors (Proppe et al. 2017; St. Clair et al. 2019) proposed that learning principles can also mitigate transportation-caused wildlife mortality. Alternatively, learning can be counterproductive to management efforts, such as in the case of habituation, which can attenuate an animal response to a repeated sensory cue (Blumstein 2016; Papaj et al. 2019). In order to achieve long-term deterrence, it is crucial to consider the capacity for learning in animals (Schakner and Blumstein, 2013).

Studying wild raccoons allows the testing of animals in ecologically relevant contexts. A limitation, especially in learning studies, is that we have no information on the animal’s background. Technological advances can aid in the study of learning by enabling individual identification, which is essential for examining repeated attempts by the same individual. Griebling and colleagues (2022) present RFID tags and machine-learning-assisted identification as candidate technologies for this challenge. Replicating the experiments in an urban context could inform us about the effect of exposure to humans on learning, where raccoons are often part of conflicts (Gehrt 2004; Curtis and Hadidian 2010). Long-term and repeated experiments will inform us about the parameters influencing the learning and memorization of new knowledge. With the integration of cognitive knowledge of target species and technological advances, new mitigation methods can be devised to reduce the severity of conflicts with troublesome individuals in a paradigm of respectful coexistence with wildlife.

## Supplementary Material

arae046_suppl_Supplementary_Video_S1

arae046_suppl_Supplementary_Video_S2

## Data Availability

Analyses reported in this article can be reproduced using the data provided by Lazure and Weladji (2014).
